# Unlocking the wasting enigma: Highlights from the 8th Cachexia Conference

**DOI:** 10.1002/jcsm.12106

**Published:** 2016-02-17

**Authors:** Nicole Ebner, Stephan von Haehling

**Affiliations:** ^1^ University Medical Center GoettingenDepartment of Cardiology and Pneumology, Innovative Clinical TrialsGoettingenGermany

**Keywords:** Cachexia, Muscle wasting, Sarcopenia

## Abstract

This article highlights pre‐clinical and clinical studies into the field of wasting disorders that were presented at the 8th Cachexia Conference held in Paris, France December 2015. This year some interesting results of clinical trials and different new therapeutic targets were shown. This article presents the biological and clinical significance of different markers and new drugs for the treatment of skeletal muscle wasting. Effective treatments of cachexia and wasting disorders are urgently needed in order to improve the patients' quality of life and their survival.

## Introduction

The development of preventive and therapeutic strategies against cachexia and wasting disorders including sarcopenia, are perceived as an urgent need by health professionals and have instigated intensive research on the pathophysiology of these syndromes.^1^, ^2^ Cachexia is characterized by progressive weight loss affecting different body compartments, particularly muscle tissue and adipose tissue, although even bone mineral content may be affected.^3^ Over the last years, the Cachexia Conference has developed to a forum for researchers from the fields of cachexia and wasting disorders. It is unique in several ways as it provides a platform for both clinicians and basic researchers to meet and discuss pathways and potential therapeutic targets as well as recent evidence from clinical trials. The 8th Cachexia Conference was held in Paris, France, from 4 to 6 December 2015 with over 500 participants from more than 35 countries attending.

### Basic Science

One interesting candidate in the pathway of muscle wasting, presented at the conference, is the tumor necrosis factor (TNF)‐like weak inducer of apoptosis (TWEAK). Kumar *et al*. (University of Luisville, Kentucky, USA) presented interesting data to the role of TWEAK‐Fn14 signalling in muscle wasting.^4^ TWEAK‐transgenic mice were produced that expressed high levels (>14‐fold) of TWEAK protein. Additionally, a TWEAK knockout mouse was used to reveal novel mediators of skeletal muscle atrophy. The authors elegantly demonstrated that the TWEAK–Fn14 system is an important target for preventing skeletal muscle wasting. The loss of myosin heavy chain in the 6‐month‐old TWEAK‐ transgenic animals indicates that the MuRF1–myosin heavy chain pathway has been activated by TWEAK–Fn14 signaling. MuRF1 is a specific E3 ligase for myosin heavy chain protein. Its activation causes the breakdown of myosin heavy chain and other components of the thick filament of the sarcomere during atrophy. To establish a TWEAK–Fn14–NF‐κB–MuRF1–myosin heavy chain protein degradation cascade, it was investigated whether the expression of TWEAK or its receptor Fn14 is affected in skeletal muscle in conditions of atrophy and hypertrophy *in vivo*. The cytokine TWEAK and its cognate receptor Fn14 are members of the TNF/TNFR superfamily and are upregulated in tumors. In recent literature, Johnston *et al*.^5^ showed that Anti‐Fn14 antibodies prevented tumor‐induced inflammation and loss of fat and muscle mass. Fn14 signalling in the tumor, rather than in the host, seems to be responsible for inducing this type of cachexia because tumors in Fn14‐ and TWEAK‐deficient hosts developed cachexia. These results indicate that Fn14 antibodies may be a promising approach to treat cachexia, thereby extending lifespan and improving quality of life in patients with cancer. Another new target for therapeutic interventions seems to be the tumour‐derived parathyroid‐hormone‐related protein (PTHrP). By using a Lewis lung carcinoma model of cancer cachexia, Kir *et al*.^6^ recently showed that PTHrP has an important role in wasting by driving the expression of genes involved in thermogenesis in adipose tissues. Neutralization of PTHrP in tumour‐bearing mice blocked adipose tissue browning and the loss of muscle mass and strength. These results demonstrated that PTHrP mediates energy wasting in fat tissues and contributes to the broader aspects of cancer cachexia. Thus, neutralization of PTHrP might hold promise for ameliorating cancer cachexia and improving patient survival. The role of the gut microbiota in therapeutic management of cancer and associated cachexia receives more and more attention in the last years. Bindels *et al*.^7^ (Université Catholique de Louvain, Brussels, Belgium) presented amazing data from two mouse models of cancer cachexia in acute leukaemia, BaF model or C26 model with subcutaneous transplantation of colon cancer cells. They analysed the role of the gut microbiota in the therapeutic management of cancer and associated cachexia and showed that the development of cancer outside the gut can impact intestinal homeostasis and the gut microbial ecosystem. They demonstrated that the gut microbial balance was altered by the presence of tumours located outside the gastrointestinal tract. Cecal *Lactobacillus* spp. levels were decreased in leukemic mice with cachexia, and the denaturing gradient gel electrophoresis analysis suggested that bacteria other than lactobacilli were affected by the disease.^8^ A microbial signature common in both mouse models including the Entero‐bacteriaceae family/Escherichia genus, Lactobacillus genus and Parabacteroides goldsteinii/ASF 519 species were presented. Interestingly, a highly significant negative correlation was found between the level of lactobacilli in the cecal content and the atrophy marker expression in the gastrocnemius muscle (Atrogin‐1, MurF1, LC3 and Cathepsin L).^9^ Moreover, they impressively demonstrated that cancer cachexia was associated with profound alterations of the gut microbiota in two animal models. Future studies should be focused on investigating the causative role of specific gut microbes and altered intestinal homeostasis in the progression of cancer and associated cachexia.

A number of elegant models were presented in order to improve our understanding of pathways involved in the wasting process. Muscle wasting has received increasing research efforts in recent years. Thus, one of the hot topics of the meeting was the investigation of biomarkers using muscle proteome and urine proteome. Skipworth *et al*.^10^ (University of Edinburgh, Edinburgh, UK) presented data from urine proteomics including one‐dimensional sodium dodecyl sulfate polyacrylamide gel electrophoresis followed by matrix‐assisted laser desorption/ionisation or liquid chromatography tandem mass spectrometry to show the protein content of urine from cachectic (>10% weight loss) (*n* = 8) and weight‐stable (*n* = 8) gastro‐oesophageal cancer patients and healthy controls (*n* = 8). The number of protein species identified in cachectic samples was greater than that identified in weight‐stable cancer and control samples. Many of the proteins identified have not been reported previously in the urine of cancer patients. Proteins identified specifically in cachectic samples included muscle (myosin species), cytoskeletal (*α*‐spectrin; nischarin) and microtubule‐associated proteins (microtubule‐actin crosslinking factor; microtubule‐associated protein‐1B; bullous pemphigoid antigen 1), whereas immunoglobulin *κ*‐light chain and zinc *α*‐2 glycoprotein appeared to represent markers of cancer. Using urinary proteomics they suggest myosin 2 as new potential marker. Thus they conclude that urinary proteomics can identify muscle‐specific and non‐muscle‐specific candidate biomarkers of cancer cachexia.

### Body composition

Most impressive data in the field of body composition and exercise were presented by Vanderbyl *et al*. (Jewish General Hospital, Montreal, Quebec, Canada)^11^ about the timing of standard exercise training, which may affect the therapeutic benefits for advanced cancer patients undergoing treatment. In this randomized, cross‐over trial, patients with advanced stage of lung and gastrointestinal cancers were included. Patients underwent a supervised Qigong training or standard exercise training twice weekly for six weeks. The surprising results showed that standard exercise training is better than Qigong training for reducing patients' feelings of weakness and their measured endurance capacity (six minute walk test). There was no difference between the effects of both training methods on symptoms and quality of life. The impact of order, and the reduced improvement in exercise function with standard exercise training in the second interval was surprising. These results may indicate that the functional benefits from standard exercise interventions are more evident when used early in the patients' trajectory.^12^ Kaeding *et al*.^13^ (University of Oldenburg, Oldenburg, Germany) presented data from whole body vibration training for the prevention of cancer cachexia during allogeneic hematopoietic stem cell transplantation. The aim of this study was to examine whether performing whole body vibration training on every second day of hospitalization in combination with classic general physiotherapy assists in stabilizing the physical capacity of patients undergoing haematopoietic stem cell transplantation. A total of 26 patients were randomly assigned to an intervention group (*n* = 13) or to the control group (*n* = 13). They showed the safety and suitability of such an intervention but whole body vibration training did not influence muscle strength in this intervention significantly. Maddocks *et al*.^14^ (King's College London, London, UK) gave an overview of assessments of skeletal muscle function including handgrip strength, quadriceps muscle strength measurement, stair climb power test and sit to go stand. He stated that “The devil is in the detail–treat seemingly simple measurement with respect.” Different techniques to measure body composition were presented during the congress including computed tomography (CT) scanning, dual energy X‐ray analysis (DEXA) and magnetic resonance imaging (MRI), D3‐creatine dilution analysis, and bioimpedance analysis (BIA). Slee *et al*.^15^ (United Lincolnshire Hospitals Trust, Lincoln, UK) demonstrated that frail older people tend to have a high degree of impaired functional ability and comorbidity and have a potential co‐existence of states such as malnutrition, sarcopenia, and cachexia. Utilization of BIA measurements as a component of the comprehensive geriatric assessment as part of gold standard clinical care is novel and may assist in the accurate assessment of nutritional and sarcopenia domains. They showed some interesting additional implications relating to prognostic potential of phase angle assessment. The mean phase angle for men was 4.7° ± 1.3° (2.4°–9.2°) and for women 4.5° ± 0.7° (2.8–6.0°). The phase angle correlated with the Mini‐Nutritional Assessment score (*P* = 0.05). BIA may represent a useful non‐invasive tool for the clinical assessment of this population group–but more research is necessary. However the most amazing method was recently descripted by Stimpson *et al*. 16 Indeed, the D3‐creatine dilution method can be applied for the determination of total body creatine pool size and, thus, skeletal muscle mass. This interesting method can directly assess skeletal muscle mass or its change, during aging, inactivity, disease, or during periods of exercise. This method takes advantage of a number of aspects of creatine biology. More than ninety percent of the total body creatine pool is found in skeletal muscle. Newly synthesized creatine from hepatic and renal sources is transported into the sarcoplasm against a large concentration gradient. 2H labeled creatine is ingested as a 30 mg capsule and distributed to skeletal muscle. Creatine is converted to creatinine and excreted in urine. Evans *et al.* (KineMed, Inc., North Carolina, USA) presented results of a clinical validation study demonstrating that the creatine dilution method is strongly associated with whole body MRI‐method. The dose of D3 creatine in adults is 30 mg and 2 mg in infants. The D3 creatine dilution method appears to be a valid non‐invasive measurement for muscle mass in infants^17^ and children. In some adults, a portion of the ingested D3 creatine label is filtered by the kidney and spilled into urine. By measuring creatine/creatinine ratio, a correction for spilled label is determined using an appropriate algorithm. Combined with dosing of ^2^H_2_O, lean body mass and muscle mass can be measured non‐invasively. Importantly the measurement of muscle mass using the creatine dilution method is not affected by shifts in body water that occur with many cachectic diseases and that sometimes poses a confounder in other measures of skeletal muscle estimations.

### Clinical trials and treatments

Loss of skeletal muscle mass and strength plays a significant pathological role in the progression of a wide variety of disorders associated with aging and catabolic conditions. Neutralization of myostatin activity results in skeletal muscle hypertrophy and prevents atrophy in adult skeletal muscle. REGN1033 is a fully human monoclonal antibody administered subcutaneously that specifically binds myostatin and blocks its function. Donahue *et al*. (Regeneron Pharmaceuticals Inc., New York, USA) presented data on the efficacy of the specific antagonist of myostatin named REGN1033. REGN1033 was tested in 253 patients with sarcopenia. They were split into four groups: (i) placebo group (*n* = 55), (ii) 100 mg REGN1033 subcutaneously every 4 weeks (*n* = 63), (iii) 300 mg REGN1033 subcutaneously every 4 weeks (*n* = 65) and (iv) 300 mg REGN1033 subcutaneously every other week (*n* = 60). Compared with placebo, all doses of REGN1033 treatment were associated with a significant increase in total lean body mass from baseline to week 12. At the higher dose (300 mg) REGN1033 treatment resulted in significant decreases in total and regional fat mass. Mean values of strength and function tests generally tended to indicate a beneficial direction with REGN1033 treatment. REGN1033 was well tolerated at all 3 doses tested. The results showed that REGN1033 increases muscle mass, force production, and physical performance outcomes in patients with sarcopenia, and preventing the loss of muscle mass. Thus, the use of antagonists of myostatin may be worthwhile in clinical testing.

Other therapeutic treatments include the selective androgen receptor modulators (SARMs). SARMs are a new class of non‐steroidal, tissue specific, anabolic agents that have the potential to increase muscle mass and improve physical function without the unwanted effects on the prostate, skin, or hair that are commonly associated with testosterone or other nonselective, synthetic anabolic steroids. Enobosarm is a nonsteroidal SARM that induces conformational changes in the androgen receptor upon binding, which selectively alters the interaction of the receptor with co‐activator and co‐repressor proteins that exist in different tissues and changes the receptor's ability to regulate gene expression.^18^, ^19^ Improvements in lean body mass and physical function were shown in a phase II, double‐blind, placebo‐controlled study of enobosarm in healthy postmenopausal women and elderly men.^18^ This study showed that both 1 mg and 3 mg of enobosarm resulted in increases in lean body mass in patients with advanced cancer, compared with baseline measurements. Crawford *et al*. (Duke Cancer Institute, Durham, USA) gave an update of recent enobosarm developments. They showed data from the International Pivotal Phase III Clinical Trials G300504 and G300505, which used enobosarm for prevention and treatment of muscle wasting in patients with non‐small cell lung cancer (NSCLC) stage III/IV at initiation of first line chemotherapy. Enobosarm (3 mg) was tested in patients with platinum and taxane‐based chemotherapy (G300504) and in patients with platinum and non‐taxane‐based chemotherapy (G300505), both against placebo. These trials confirmed that patients with advanced NSCLC have severe muscle loss and physical function impairment at diagnosis that decline further with platinum‐based chemotherapy treatment. Enobosarm treatment was associated with an increase in lean body mass compared with a decline in the placebo group in both studies. In G300504, enobosarm treatment was associated with better stair climb performance compared with placebo but not so in G300505. Lean body mass response was associated with an improvement of both physical function and survival, as well as maintenance of quality of life.

Ghrelin and ghrelin receptor agonists are being explored for their potential impact on clinical conditions such as anorexia and cancer cachexia. These compounds have been found to have clinical attributes, which make them well‐suited for the treatment of cachexia.^20^ Ghrelin is a potent orexigenic hormone and its administration increases body weight by enhancing appetite, muscle, and fat accrual.^21^ Fearon *et al*. (Western General Hospital, Edinburgh, UK) presented data from the Safety and Efficacy of Anamorelin HCl in Patients With Non‐Small Cell Lung Cancer‐Cachexia (ROMANA 1 and 2) trials with ghrelin receptor agonist anamorelin. Anamorelin is currently in phase III development, with two parallel trials in NSCLC cachexia recently completed^22^ ROMANA‐1 (NCT01387269) and ROMANA‐2 (NCT01387282). In addition, patients from these studies had the option of continuing treatment in a 12‐week safety extension study^23^ ROMANA‐3 (NCT01395914). Takahashi *et al*. (RaQualia Pharma, Japan) identified a new potent ghrelin receptor agonist, RQ‐00433412. RQ‐00433412 increased growth hormone release in mice and rats, increased body weight in normal mice, attenuated decrease in body weight and increased food consumption in cisplatin treated rats. They showed attenuated decrease in body weight and muscle mass, without changes of food consumption in AH‐130 hepatoma rat cachexia model. Ghrelin and ghrelin receptor agonists are potential useful in cancer anorexia/cachexia.

### Conclusions

From basic science new therapeutic targets were shown including the TWEAK–Fn14–NF‐κB–MuRF1– myosin heavy chain protein degradation cascade and the neutralization of tumour‐derived parathyroid‐hormone‐related protein, as well as the influence and the role of the gut microbiota in the therapeutic management of cancer and associated cachexia. Effective Treatments were REGN1033, Ghrelin and ghrelin receptor agonist anamorelin and enobosarm. Many new hopes have appeared on the cachexia treatment horizon recently, and several drugs have shown to increase muscle mass but failed to translate this effect into increased strength. Other substances remain in preclinical testing, but we are awaiting clinical testing to commence soon for RQ‐00433412. There is further need for attractive biomarkers as therapeutic target. Urine proteomics identified new potential biomarkers for example myosin species, proteins of the cytoskeletal (*α*‐spectrin; nischarin) and microtubule‐associated proteins. For non‐invasive measurement of skeletal muscle mass the D3‐creatine dilution method can be applied repeatedly to measure total body creatine skeletal muscle mass change in longitudinal studies.

## Conflict of interest

The authors N.E. and S.vH. declare that they have no conflict of interest.
